# Explainable AI for effective management of urban heat sources

**DOI:** 10.1038/s41598-025-24305-z

**Published:** 2025-11-18

**Authors:** Maciej Sabal, Tomasz Danek, Mateusz Zaręba, Elżbieta Węglińska

**Affiliations:** 1 Department of Metalworking and Physical Metallurgy of Non-Ferrous Metals, Faculty of Non-Ferrous Metals, AGH University of Kraków, Kraków, Poland; 2 Department of Geoinformatics and Applied Computer Science, Faculty of Geology, Geophysics and Environmental Protection, AGH University of Kraków, Kraków, Poland

**Keywords:** Particulate matter, Machine learning, Air pollution, PM2.5, Programs, Heating sources, Computational science, Scientific data, Environmental sciences

## Abstract

Many cities around the world face the challenge of polluted air, even when implementing restrictive heat source policies. Analyses of air flows often reveal that particulate matter is primarily carried from neighboring areas. Programs are frequently introduced to encourage the replacement of old, inefficient heat sources with cleaner alternatives, though the use of solid fuels is not always prohibited. In this paper, we analyze the case of Kraków as a testbed due to its unique conditions: strict regulations on heat sources within the city, a lack of such policies in surrounding municipalities, and a dense network of low-cost sensors (LCS) monitoring air quality. Additionally, precise data on the number and type of heating sources in municipalities neighboring Kraków is available. Moreover, tax revenue per capita for these municipalities was incorporated, providing additional socioeconomic context for emissions management. The purpose of the paper is to assess the impact of neighboring municipalities on particulate matter concentrations in Kraków, considering heat source replacement programs and tax revenue data. An analysis of program impacts on air quality is made, and a revision of the project formula addressing sustainability is recommended. To achieve these objectives, a Spatiotemporal Explainable AI (XAI) with Dynamic Time Warping (DTW), Random Forest (RF), and direct interpretability techniques, including H statistics and Accumulated Local Effects (ALE), were applied. This approach identified emission patterns which, combined with emissions data, facilitated developing a model supporting data-based management. The results highlight the necessity of integrating socioeconomic factors to design sustainable air quality policies.

## Introduction

Among the major quality of life issues is clean air. With the rapid economic development associated with globalization, increasing energy demand and large-scale transportation, emissions causing air pollution are increasing. The problem is particularly important in urban areas^1^ Cities like Kraków face challenges similar to global metropolises such as Delhi and Shanghai, where air pollution levels are driven by a combination of industrial emissions, transportation, and energy demands (e.g^2–4^. Depending on location, terrain, demographics, sources of air pollution vary widely, both natural and resulting from human activity (e.g^5^.

The substances most often emitted into the atmosphere as a result of commercial activities include sulfur dioxide, nitrogen oxides, particulate matter, volatile organic compounds, heavy metals, among others^6^. Nitrogen oxides, which are emitted from road transportation, as well as the burning of fossil fuels, are a particularly important problem. Nitrogen oxides pose a very high health risk^7,8^. This research focuses mainly on particulate matter (PM) because studies show that it is a significant factor contributing to the development of neurodegenerative diseases such as Alzheimer’s. This could become a major social issue in an aging society like Krakow’s future population^9^.

The phenomenon of air pollution is one that has been of interest to policymakers and researchers alike for several decades. In the mid-1990s, sources of urban air pollution in Leeds (UK) were analyzed. The study found that volatile organic compounds emitted by vehicles accounted for nearly half of all emissions^10^. A later analysis in and around Berlin showed that pollution is much higher in the urban area than in suburban areas^11^. Since then, many studies have pointed to agglomerations as places where air pollution is a major problem^12,13^. As a result of this problem, more and more countries around the world are working to formulate effective policies for managing urban air quality.

Air pollution is a major social problem, but also a health problem. The link between exposure to polluted air and diseases, including those related to the respiratory system, cardiovascular system or cancer, has been proven^14,15^. Air pollution also has fatal effects, and is considered a major environmental factor contributing to premature deaths^16^. It is estimated that up to 307,000 premature deaths in Europe in 2019 were caused by exposure to PM2.5 and PM10. In addition, about 40,000 premature deaths were attributed to chronic exposure to nitrogen dioxide^17^. In the European Union, the highest mortality associated with polluted air is found in northern Italy, southern Poland and eastern Czech Republic^18^.

Poland is among the countries struggling with the problem of polluted air. It is home to 36 of the most polluted cities in Europe^19^. The issue is of concern to both central and regional authorities and residents themselves. A 2019 survey found that according to 62% of Poles, air pollution is one of the top three environmental problems, while the global average is 35%^20^.

An example of a city that faces the problem of polluted air is Kraków. The city is located in the southern part of Poland, east of industrialized Upper Silesia and northeast of the Czech Republic. Southern Poland, like the eastern Czech Republic, is characterized by high levels of anthropogenic emissions, related to coal combustion during the winter months, but most importantly, unfavorable climatic conditions in the area^21,22^. Southern Poland, including Kraków, as well as Ostrava in eastern Czech Republic, were centers of heavy industry for decades. In Kraków, since the 1950s, it was primarily the metallurgical industry. The situation began to improve in the 1990s, due to greater awareness of the problem, as well as technological changes that reduced emissions^23^. However, the problem has not been completely solved.

In 2018, the World Health Organization (WHO) ranked Kraków 8th on its list of the 50 most polluted cities in the European Union^24^. It should be noted that the city authorities introduced advanced policies to combat air pollution. In 2019, a total ban on heating buildings and preparing meals with solid fuels, i.e. coal, wood or biomass, were introduced^25^. While these radical measures have improved air quality, pollution takes an excessive toll during the winter months. Power plant operations, low emissions and traffic are responsible. The decisive problem that determines air quality, however, is related to Kraków’s geographic location and, inextricably linked to the transport of air masses from neighboring municipalities that do not have restrictive regulations related to air protection^5^. The city’s location in the Vistula River valley causes air quality in Kraków to deteriorate rapidly during the autumn and winter, when heating with solid fuels takes place in neighboring communities. Although the authorities of the Małopolska Region, as well as the central government, are offering a number of solutions for modernizing heat sources, the process is having a limited effect.

Traditional methods of creating management models rarely take into account advanced spatial and temporal analyses of the phenomena affecting them. The authors, thanks to their access to two valuable data sources—information on particulate matter, as well as precise information on heat sources—have for the first time compiled them for an agglomeration area. This paper not only provides an in-depth and precise analysis of the quantity and quality of heat sources, but also correlates these changes with atmospheric factors in an advanced spatial-temporal framework. Using ML techniques such as unsupervised clustering, PCA and time series analysis, this study contextualizes models and analyses within the framework of regulatory changes. It demonstrates the synergy between the three components as inseparable elements for researchers seeking to implement regional governance policies. A typical public management problem is the separate treatment of individual data and subject areas, which is sometimes the result of the division of responsibilities among different administrative units. This, in turn, makes it difficult to collate these data and thus to analyze them holistically. The analysis carried out in this study includes an approach that combines different perspectives, so it can make a valuable contribution to the formation of public policies based on the principles of sustainable development. The authors used a unique source of information, which is a database of household heating devices, which made it possible to match data from air quality sensors with types of stoves. On the basis of observation and analysis, it was possible to determine the behavioral patterns of residents of particular areas in municipalities around Krakow. In addition, the paper presents information on municipalities around Krakow in the context of tax revenue per capita, which made it possible to link the wealth of these municipalities to air quality. The findings of the publication can be a valuable source for regional authorities on how to take the social aspect into account so that public policy on air quality is sustainable and takes into account various aspects, including those related to household wealth.

The main purpose of this paper is to assess how neighboring municipalities influence particulate-matter concentrations in Kraków—with particular attention to heat-source replacement programs and local tax-revenue data—and, because our evidence is largely observational, we present these findings as strong associations rather than definitive relationships.

### Projects and programs for improving air quality in Kraków

Measures to protect air quality in Kraków, as well as throughout Poland, involve the implementation of programmes. In the case of Kraków, a very important step was the adoption in 2011 of the Low Emission Reduction Programme for the City of Kraków^26^. This programme provided subsidies for residents who decided to replace their solid fuel heating systems. The subsidy covered the costs of purchase and installation. Both individuals and small businesses were eligible for the programme. The programme was updated in 2014 and 2015.

The document that had a key impact on air protection policy is the resolution of the Małopolska Provincial Assembly of January 2016, which, as of 1 September 2019, introduced a ban on the use of solid fuels (coal, wood and other types of biomass) in Kraków, commonly known as the anti-smog resolution^27^. At the same time, in addition to the ban on the use of solid fuels, Kraków adopted a protective programme of the Municipal Social Welfare Centre in Kraków, consisting of subsidising incresed heating costs for people with the lowest incomes^28^.

The city authorities therefore approached the problem methodically, by implementing programmes which, according to the literature, are defined as a group of interrelated projects carried out in a coordinated manner, using the same resources and managed by a programme organisation in order to achieve one or a number of strategic objectives^29,30^. The programmes implemented by the Kraków authorities had specific objectives, the result of which was to eliminate solid fuel heating sources. It is worth noting that programme management uses project management methods, i.e. they are time-limited undertakings^31,32^. Programmes are more complex than projects, but in both cases, the key issue is the need to achieve objectives within a specified time frame^33^. In the case of programmes aimed at improving air quality, the issue of temporariness is a key element. This stems from the logic of project and programme management in the European Union (EU), whose regulations have a significant impact on environmental policy. EU programmes are implemented in cycles lasting several years, which end with an evaluation^34^. This allows for effective monitoring and analysis of how the money spent translates into assistance in a given area.

However, this also has consequences for the form of support for residents and local governments, because the programme must be implemented within a specified time frame and, importantly, the funds allocated by the EU from the so-called Multiannual Financial Framework must be spent. The authorities in Kraków, which have benefited from EU programmes, have planned their support in such a way as to achieve the objective (eliminating solid fuel heating sources) within a specified time frame. Local governments in EU countries, including Poland, have mastered the tools, platforms and interrelationships between EU, national and local programmes that make up the complex ‘world of EU-funded projects^35^. However, in the case of Kraków, costly measures in the form of air quality improvement programmes have still not eliminated this problem in the city. An important challenge for the city is the issue of air quality and heat sources in the surrounding municipalities.

### Air quality around Kraków

**Fig. 1:**
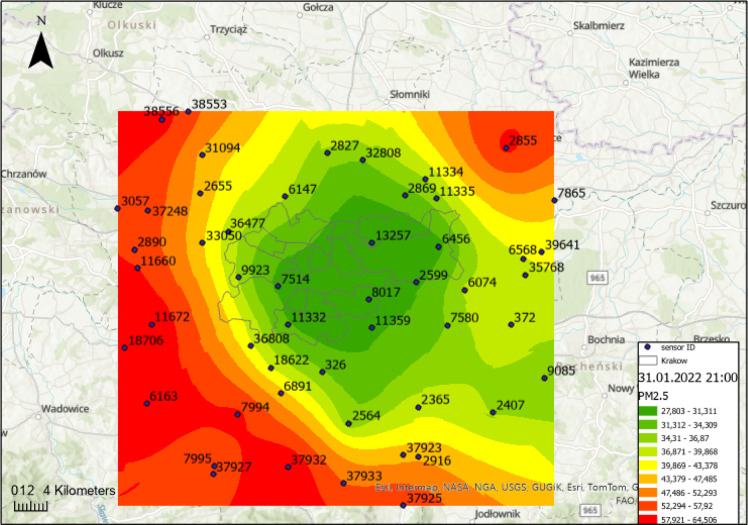
Spatial distribution of PM2.5 concentrations around Kraków area on January 31, 2022, at 21:00, calculated using the ordinary kriging method (own study, ArcGIS Pro 3.0.3, ESRI).

^5^ provides a comprehensive analysis of PM concentrations using geostatistical methods to assess the spatial distribution and variability of air pollutants in the study area. Conducted in early spring during the COVID-19 pandemic, the research focused on observing pollutants mainly from the combustion of solid fuels, as reduced car traffic minimized background pollution. Their study demonstrates the effectiveness of geostatistical methods and the Geographically Weighted Regression model in identifying areas of varying PM emissions and distinguishing pollutants from combustion versus other anthropogenic sources. The analysis revealed that the impact of meteorological factors on PM concentration is complex, with wind direction and speed, and air pressure playing varying roles depending on the conditions and phases of pollutant flow. Their research showed that the relative temperature perception had a direct impact on PM concentrations.

Kraków is among the cities with a total ban on the burning of solid fuels. However, despite this regulation, the issue of smog remains highly prevalent due to the city’s geographical location. Research has shown that the primary factors contributing to this situation are the city’s topography (depression associated with the Vistula River valley) combined with generally weak predominantly western winds^5,36–38^. Additionally, the lack of similar restrictions in neighboring towns amplifies the problem. The Fig. 1 presents a typical example of the PM2.5 distribution in Kraków, calculated using the ordinary kriging method, depicting the concentration levels on January 31, 2022, at 21:00, with a distinct formation of the characteristic “bagel” pattern. In the subsequent hours, the pollution will move into Kraków and accumulate there. This phenomenon is both recurrent and highly characteristic for the region.

Spatiotemporal air pollution pattern was analyzed using machine learning and hot spots analysis^38^. In their analysis clustering was performed for the average and maximum concentrations of PM10. For maximum concentrations the “Kraków bagel” pattern was observed. The topography around Kraków appears to play a key role in shaping the distribution of these maximum concentrations. Kraków itself emerged as a unique cluster for maximum concentrations, standing apart from the surrounding clusters that extend outward from the city. For average concentrations, Kraków and its southeastern area formed a single cluster, while the northwestern region belonged to a different cluster, reflecting the contours of the terrain. It is also important to note that Kraków experiences six thermal seasons defined by average daily temperatures according to Guminski’s method^39^ but data on particulate concentrations suggests that only two primary seasons, warm and cold, can be distinguished^40^.

## Materials and methods

### Programs related to air protection in Kraków

The Low Emission Reduction Program for the City of Kraków was financed in part with funds from the Regional Operational Program of the Małopolska Region for 2014–2020. Thanks to the program, the city’s residents were able to replace their heat sources with emission-free ones. This allowed them to prepare for the ban on solid fuels introduced in 2019. At the same time, between 2011 and 2022 and 2023–2026, the City of Kraków provided support to the city’s poorest residents under the Local Shield Program^28^. The support consists of subsidies for residents whose incomes fall below a certain threshold. The subsidies are intended to compensate for higher residential heating costs.

The Małopolska region has a resolution restricting the use of solid fuel stoves to those appliances that meet eco-design standards^41^. The program is being implemented in stages; the most emission-intensive devices should have been eliminated by May 1, 2024, while solid fuel devices with the best characteristics (including, among others, coal stoves and fireplaces) can still be used. In the case of the City of Kraków, a subsidy program for replacing appliances was launched^26^. In the case of Małopolska, there is no separate program for the region, but residents of the province can benefit from the nationwide Clean Air Program, launched in 2020^42^. This program aims to improve air quality and reduce greenhouse gas emissions by replacing heat sources and improving the energy efficiency of residential buildings. This program is open to residents nationwide and is expected to run until 2029.

Although the scale of the Clean Air Program is very large, as it assumes the replacement of 3 million heating devices throughout Poland, people who want to take advantage of the subsidy must put up a relatively high own contribution. The program is frequently updated, at the beginning of its implementation it was possible to receive support for solid fuel stoves, although gradually the Polish authorities are phasing out this solution. A popular device that is purchased under the program is a heat pump. These devices, in turn, are dedicated to buildings with adequate thermal insulation, which, while also eligible for support, requires a high contribution. However, the program is not accompanied by a sheltering measure for lower-income families.

### Kraków agglomeration

The case analyzed in this study concerns the Kraków and neighbouring municipalities, most of which border the city. It concerns the following municipalities: Liszki, Zabierzów, Wielka Wieś, Zielonki, Michałowice, Kocmyrzów-Luborzyca, Igołomia-Wawrzeńczyce, Koniusza, Niepołomice, Biskupice, Wieliczka, Świątniki Górne, Mogilany, Skawina and Czernichów. These municipalities (without Koniusza), together with Kraków, form the Kraków Metropolis Association, which provides a platform for cooperation on joint ventures and projects in the fields of environmental protection, culture, transportation and infrastructure, among others^43^. According to research^5^, the state of the air in these municipalities has a decisive impact on the PM situation in Kraków.

Currently, only Kraków has a total ban on solid fuels. In the other municipalities of the Kraków Metropolis, this ban does not apply. Residents of these municipalities can benefit from subsidies under the Clean Air Program, as well as from local support programs offered by individual municipalities. These programs were introduced, among other things, to help residents of the municipalities comply with the resolution limiting the use of solid fuel stoves^41^. These programs offered relatively little support, and in many cases, it was possible to subsidize the purchase of a solid fuel stove provided it met eco-design standards.

In Poland, as of July 1, 2022, every homeowner is obliged to report the heating source to the Central Building Emission Register, thanks to which the information is collected in a government database^44^. Data on the number of heating sources in buildings is made available to representatives of universities and research units, providing information from individual municipalities of the Kraków agglomeration. According to the survey form, solid fuel heat sources could be self reported as:

- Solid fuel boiler (coal, wood, pellets or other type of biomass) with manual fuel feed / backfill.
- Solid fuel boiler (coal, wood, pellets or other type of biomass) with automatic fuel feeding.
- Fireplace/air heater for solid fuel (wood, pellet or other type of biomass, coal).
- Tiled stove for solid fuel (coal, wood, pellets or other type of biomass).
- Stove/coal kitchen.

Table 1 Indicates the number of solid fuel heat sources in the municipalities of the Kraków agglomeration. The table also indicates the number of inefficient solid-fuel heat sources that were eliminated in 2017–2023. During this period, more than 95,000 boilers and stoves using solid fuels were eliminated under various programs. The replacement heating device became primarily gas boilers, but also connection to the district heating network, low-emission solid fuel boilers and electric heating. This means that although in the municipalities in question a solid-fuel heating source, i.e. One that does not Meet the appropriate class, was eliminated, it was replaced by another solid-fuel stove. This data was collected by the marshal’s office of the Małopolska region on the basis of reports from municipalities^45^.

**Table 1:** Number of solid fuel heat sources in municipalities of the Kraków agglomeration and number of inefficient solid fuel heat sources eliminated in l. 2017–2023 source: general office of Building Control, 2024; Malopolska Region, 2024.

Municipality	Number of solid fuel heat sources, as of 31.12.2023	Number of total heat sources in operation, as of 31.12.2023	Number of inefficient solid fuel boilers eliminated between 2017 and 2023
Liszki	3486	8554	1464
Zabierzów	5615	16,675	1345
Wielka Wieś	2687	8098	818
Zielonki	3758	12,715	1044
Michałowice	2543	6295	764
Kocmyrzów-Luborzyca	3239	8021	855
Koniusza	1809	3166	607
Igołomia-Wawrzeńczyce	1618	3276	560
Niepołomice	5227	15,455	1095
Biskupice	2277	4899	457
Wieliczka	9669	26,434	2163
Świątniki Górne	1995	3628	587
Mogilany	3251	7841	542
Skawina	5325	8766	2610
Czernichów	4802	7957	714

**Table 2:** Tax revenue per capita of the municipality for 2022. Source: Ministry of Finance, 2024.

Municipality	Tax revenue per capita in PLN
Liszki	2 685,42
Zabierzów	3 832,12
Wielka Wieś	4 254,88
Zielonki	3 557,40
Michałowice	2 913,50
Kocmyrzów-Luborzyca	2 224,16
Koniusza	1 604,06
Igołomia-Wawrzeńczyce	1 901,79
Niepołomice	3 829,83
Biskupice	1 858,05
Wieliczka	2 662,21
Świątniki Górne	3 031,54
Mogilany	3 438,50
Skawina	3 107,76
Czernichów	2 174,30

The database is a unique source of data that not only provides insight into the distribution of types of household heating sources, but also compiles this information with other data to track broader trends. Table 2 provides information on tax revenue per capita of the municipality (in PLN currency) for 2022. This data is collected by the Ministry of Finance for the purpose of calculating subsidies for individual municipalities. On this basis, the level of wealth of individual municipalities can be estimated.

### Data

Air quality monitoring in Poland follows EU law, mainly Directive 2008/50/EC on Ambient Air Quality^46^. Nearly 6% of the almost 5000 reference stations across the EU are located in Poland, but Krakow (zone PL1201) has only a few reference monitoring PM10 and PM2.5 stations^36^. Polish standards mirror EU limits: 25 µg/m³ annually for PM2.5 and 50 µg/m³ daily for PM10, measured according to PN-EN 12,341 (manual) and PN-EN 16,450 (automatic).

**Fig. 2:**
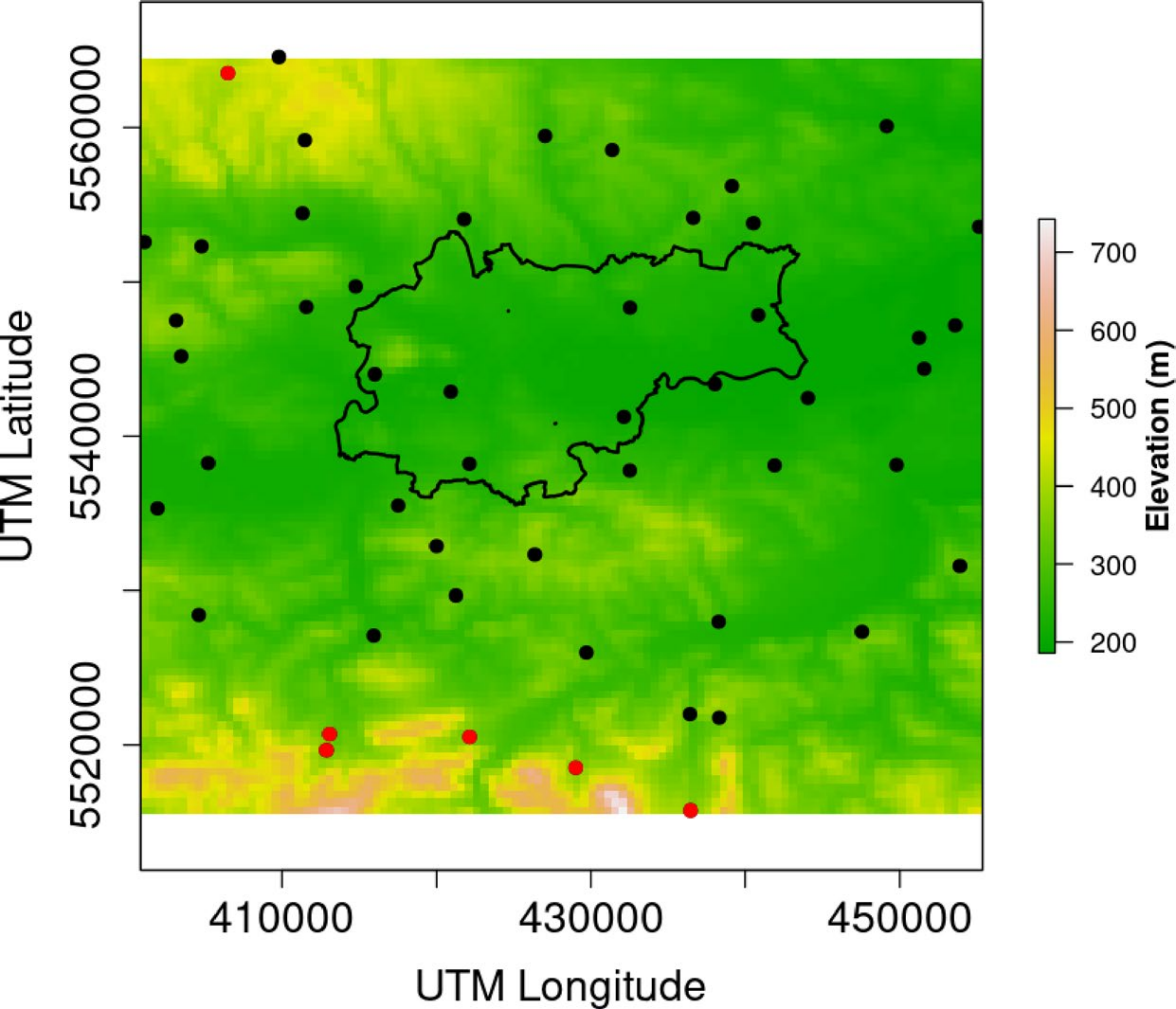
Locations of sensors mapped against the elevation profile of the Kraków area, with the boundaries of the urban agglomeration indicated. Red dots mark the locations of sensors exhibiting the old furnace pattern (see text), own study^54^.

Reference stations provide precise results but are too scarce for detailed spatial studies. Low-cost sensors (LCS) are increasingly used, though the EU does not yet recognize them officially due to weather-related biases^47^. Due to the fact that the typical LCS measurement is carried out using the phenomenon of light scattering, it is particularly sensitive to changes in humidity^48^. The input dataset included hourly PM2.5 measurements collected from quasi-regularly distributed Airly LCS, providing a reliable basis for spatial analysis. Sensor locations were chosen to capture air quality variations across different parts of Kraków and its surroundings. In studied area Airly sensors have shown performance close to reference stations, enabling geospatial analysis, though uncertainties remain—especially at extreme concentrations or under variable humidity and temperature, requiring calibration for reliable use, which is in line with scientific recommendations^49,49^. Airly sensors’s accuracy for PM2.5 is 10 µg/m^3^ in the range 0–100 µg/m^3^, 10% in the range 101–500 µg/m^3^, and 20% over concentration 500 µg/m^3^^50^. Airly provides data after calibration and corrections. Dense networks of LCS combined with geospatial methods help trace both spatial and temporal patterns, clarifying how emissions from nearby towns affect Krakow’s air^51^. The machine learning pipeline included a detailed analysis of missing values and outliers to ensure data integrity. Particular care was taken during outlier detection and removal, as rare smog events, often considered outliers, are critical for understanding PMx patterns^52^. The goal was to avoid discarding valuable data from these events. A machine learning model was used to handle missing data, relying on measurements from nearby LCS stations and the specific characteristics of the affected sensor. By incorporating both nearby sensor data and local factors, the model ensured that the imputed values closely reflected actual air quality conditions, minimizing uncertainties in the clustering process. Please note that the share of missing values never exceeded 5% for any individual sensor. For each station we therefore trained a station-specific Random-Forest regressor on the observed PMx values, hourly meteorological covariates retrieved from Open-Meteo^53^ like temperature, relative humidity, pressure, precipitation, wind, cloud cover etc. and two cyclic time features (hour-of-day, hour-of-year). The resulting R2 indicated a consistently good fit (> 0.9).The dataset includes hourly data for PM2.5, year hour, day hour, temperature at 2 m, relative humidity at 2 m, dew point at 2 m, apparent temperature, surface pressure, precipitation, rain, snowfall, cloud cover, wind speed at 10 m, wind direction at 10 m, soil temperature from 0 to 7 cm, soil moisture from 0 to 7 cm, latitude, longitude, date, and sensor ID. PM2.5 data were obtained using the Airly API^50^, while meteorological data were sourced from NOAA through the OpenMeteo API^53^. The spatial distribution of the sensors and the study area is shown in Fig. 2.

### Machine learning

To investigate the relationship between PM2.5 levels with external factors, first Principal Component Analysis (PCA) was applied to identify possible strong patterns within PM data. Random Forest (RF)^55^ models were then used, focusing on relevant predictors. Their importance was assessed using permutation methods, and the H-statistic^56^ evaluated interactions between primary predictors and other factors. Finally, Accumulated Local Effects (ALE)^57^ method was used to show nonlinear influence of main predictors on PM2.5 levels, highlighting variations in PM response to model features.

Unsupervised machine learning is effective in clustering data particularly when input variables, like concentrations, reflect complex interactions between meteorological factors, seasonality, time of day, human activity, and economic influences. It helps identify patterns and groupings without labeled data. Clustering is a technique that groups data by similarity, often requiring pre-set cluster numbers and domain knowledge for optimal results. Traditional clustering methods, like K-means with Euclidean distance, are not directly applicable to time-series data, such as PM levels. This study uses K-means with Dynamic Time Warping (DTW)^58^ for spatial and temporal clustering of complex datasets, minimizing within-cluster distances while maximizing distances between clusters, and balancing accuracy and efficiency.

**Fig. 3:**
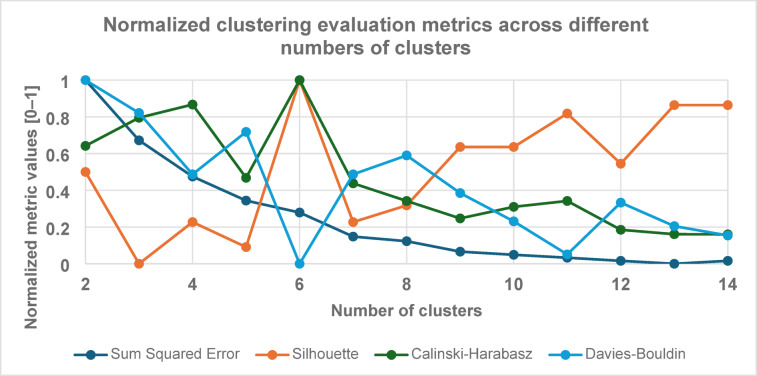
Normalized clustering evaluation metrics across different numbers of clusters.

To determine the optimal number of clusters, several metrics—Silhouette Coefficient^59^, Davies-Bouldin Index^60^, and Calinski-Harabasz Index^61^—were evaluated alongside domain insights. This approach led to selecting six clusters (see Fig. 3), enabling long-term pollution trends to be visualized on a single map. Each cluster groups sensors with similar annual PM emission patterns, regardless of geographic proximity.

## Results and discussion

### Spatial and economic patterns

**Fig. 4:**
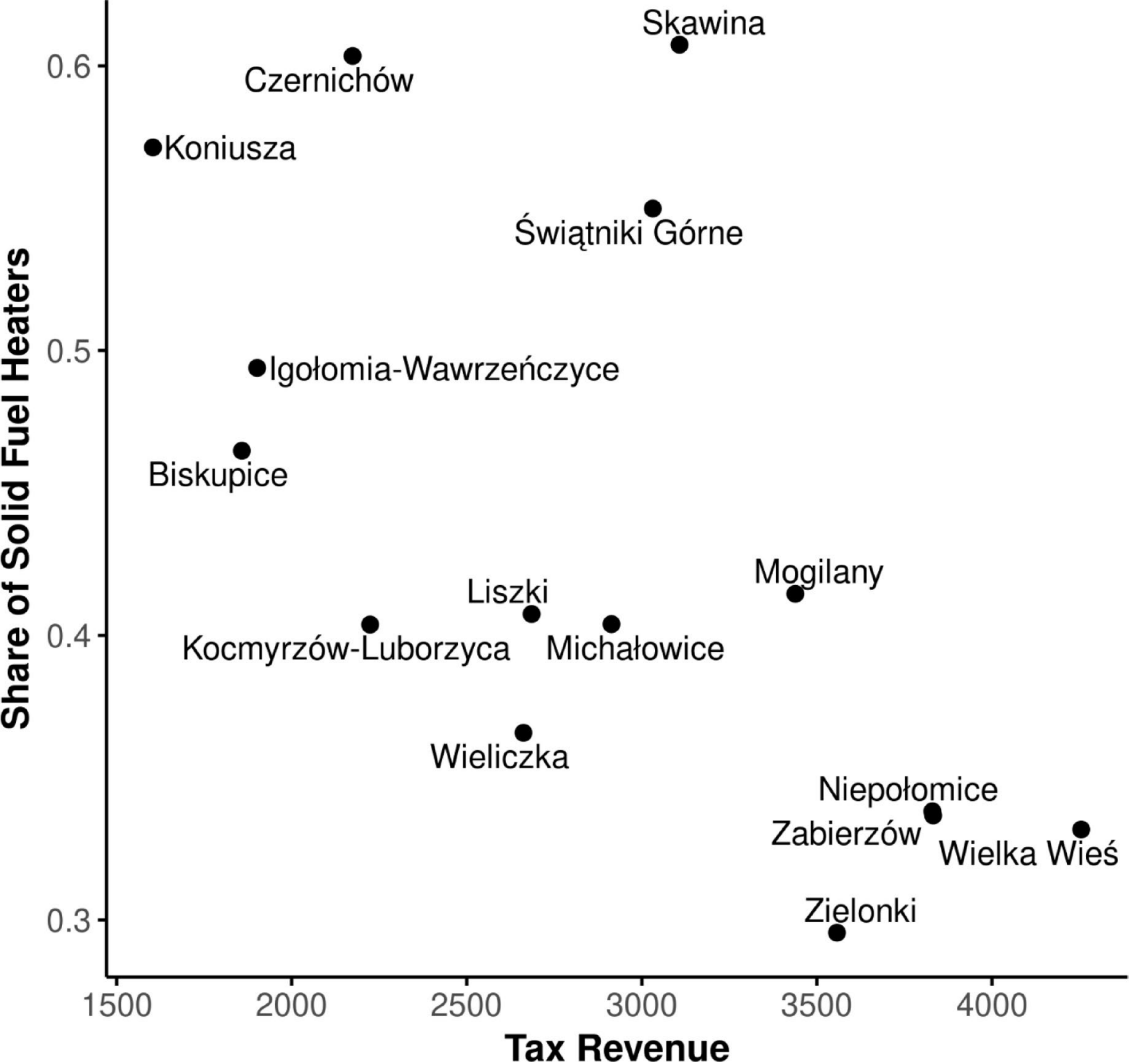
Relationship between tax revenue and the share of solid fuel heaters in municipalities surrounding Kraków.

**Fig. 5:**
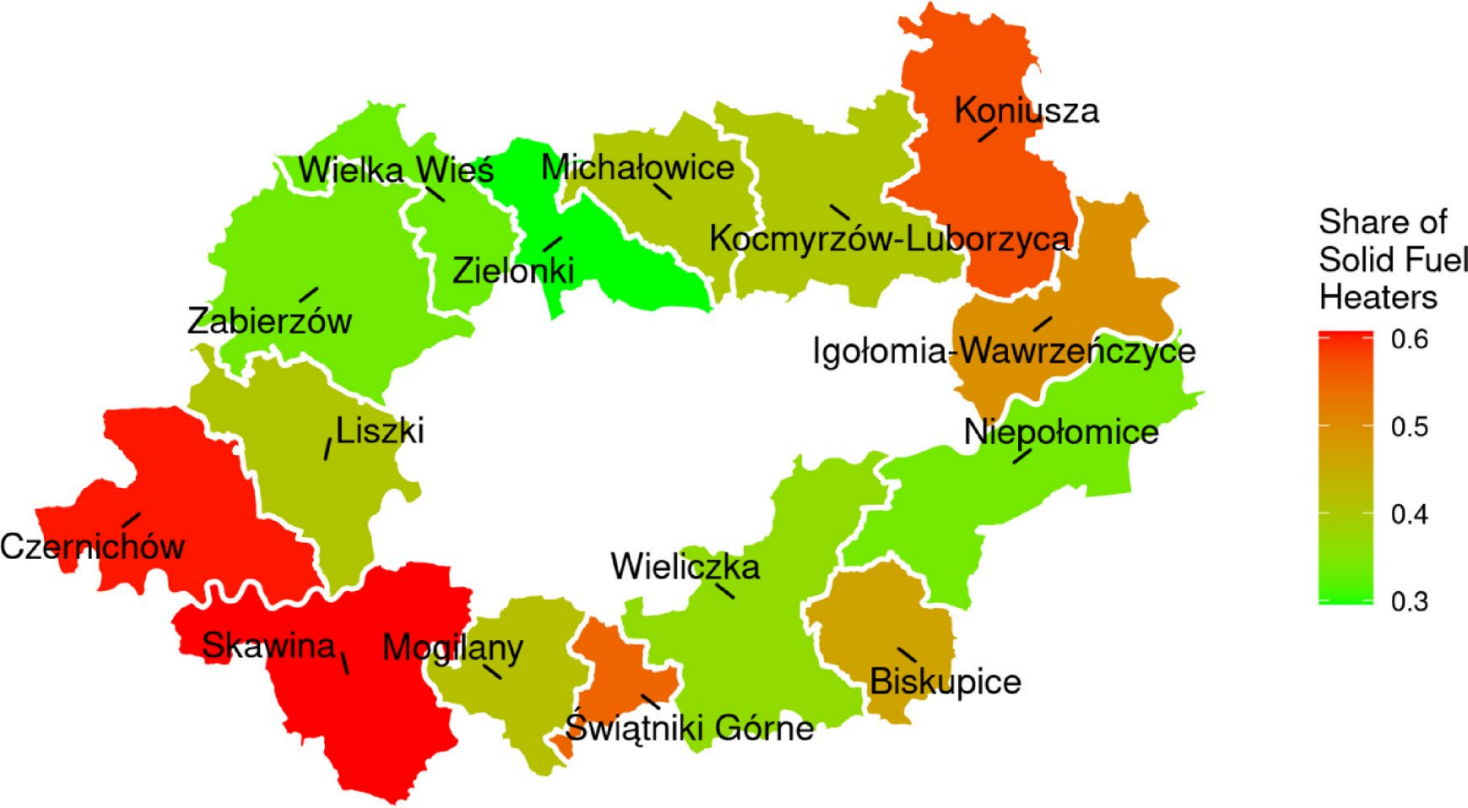
Spatial distribution of solid fuel heaters share in municipalities around Kraków, own study^54^.

Heating patterns across municipalities neighboring Kraków are influenced by both economic and spatial factors, reflecting disparities in household energy sources and their distribution. Figures 4 and 5 show the dependencies related to heating in both economic and spatial contexts. Figure 4 visualizes the relationship between municipal tax revenue and the proportion of households using solid fuel heaters for municipalities in the Kraków region. The figure shows a trend, where municipalities with higher tax revenues generally exhibit a lower share of solid fuel heaters. This relationship underscores the socio-economic disparities between municipalities, which could inform targeted policy interventions to reduce air pollution. Figure 4 illustrates the proportion of households relying on solid fuel heaters within municipalities surrounding Kraków. This visualization highlights the high geographical variability in heating sources and localizes municipalities with the highest dependency on solid fuel heaters, which are key targets for emission reduction strategies. This interplay between economic conditions and spatial variability is critical for designing effective and targeted policies to address air pollution in the Kraków region and is a necessary background for more in-depth analysis.

Based on the spatial distribution and economic trends, municipalities located directly west of Kraków, such as Czernichów and Skawina, should be prioritized in efforts to reduce the number of solid fuel heaters. These areas are not only characterized by a high share of such heaters but also lie in the predominant path of westerly winds, which increases their potential impact on Kraków’s air quality. Conversely, municipalities with increased share of solid fuel heaters located to the east or south, such as Koniusza or Świątniki Górne are probably less critical under the wind conditions, as their emissions are less likely to affect Kraków directly. But these results are based solely on data concerning heating sources and tax revenues. True understanding of the complexity of air pollution requires more advanced methods of data analysis. To effectively communicate these findings to both the public and policymakers, it is important to explain the air pollution problem both spatially and temporally. Advanced techniques are crucial for uncovering complex patterns, but it is equally important to make these insights comprehensible by using methods such as explainable AI (XAI). Without clear and transparent communication, efforts to promote heating sustainability may face significant resistance.

### Variables impact on model results

The purpose of this section is to present relevant observations related to PM2.5 concentrations in Kraków and adjacent municipalities. These observations take into account issues related to meteorological conditions and behavioral patterns. To create the model, a typical week of the heating season was considered, with diurnal fluctuations, a weekly trend, a prominent weekend, but also an anomaly due to wind.

Fifty-two sensors were located around the Kraków area. For this test data were collected for the week from Tuesday, March 15, to Monday, March 21, 2022, to better capture the effects of the weekend. All calculations were performed in R using the party and imb packages^62,63^.

**Fig. 6:**
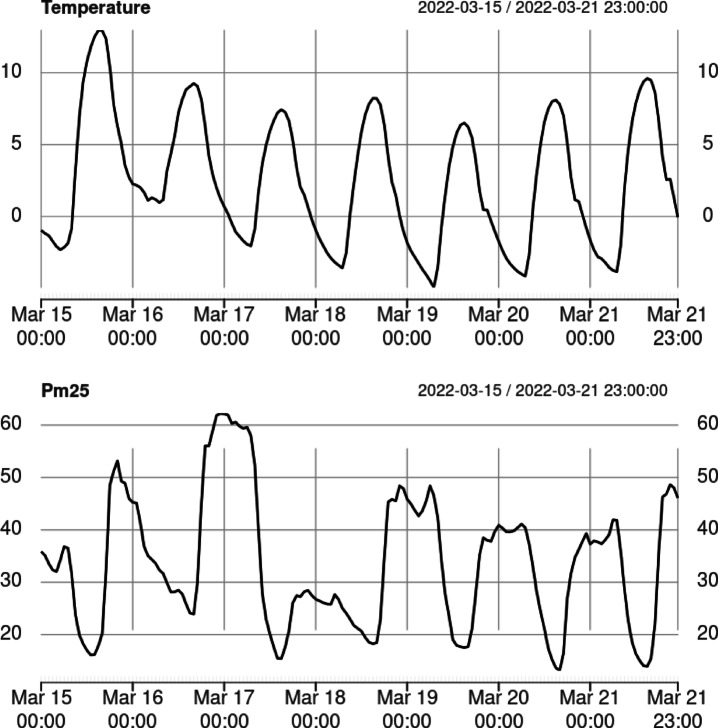
Average PM2.5 concentrations and average temperature observed across the entire study area over the analyzed period.

Figure 6 presents the average PM2.5 concentrations alongside the average temperature across the study area. It is important to note that there is no direct correlation between temperature and PM2.5 levels. Additionally, the decrease in particulate matter concentrations during the night of March 17 to 18 is also not related to temperature fluctuations at all (in fact this effect was caused by stronger than usual winds). This clearly indicates that the relationships in the data are more complex than a simple connection between cold temperatures and heating needs, and model analysis of PM2.5 concertation requires a more sophisticated approach.

**Fig. 7:**
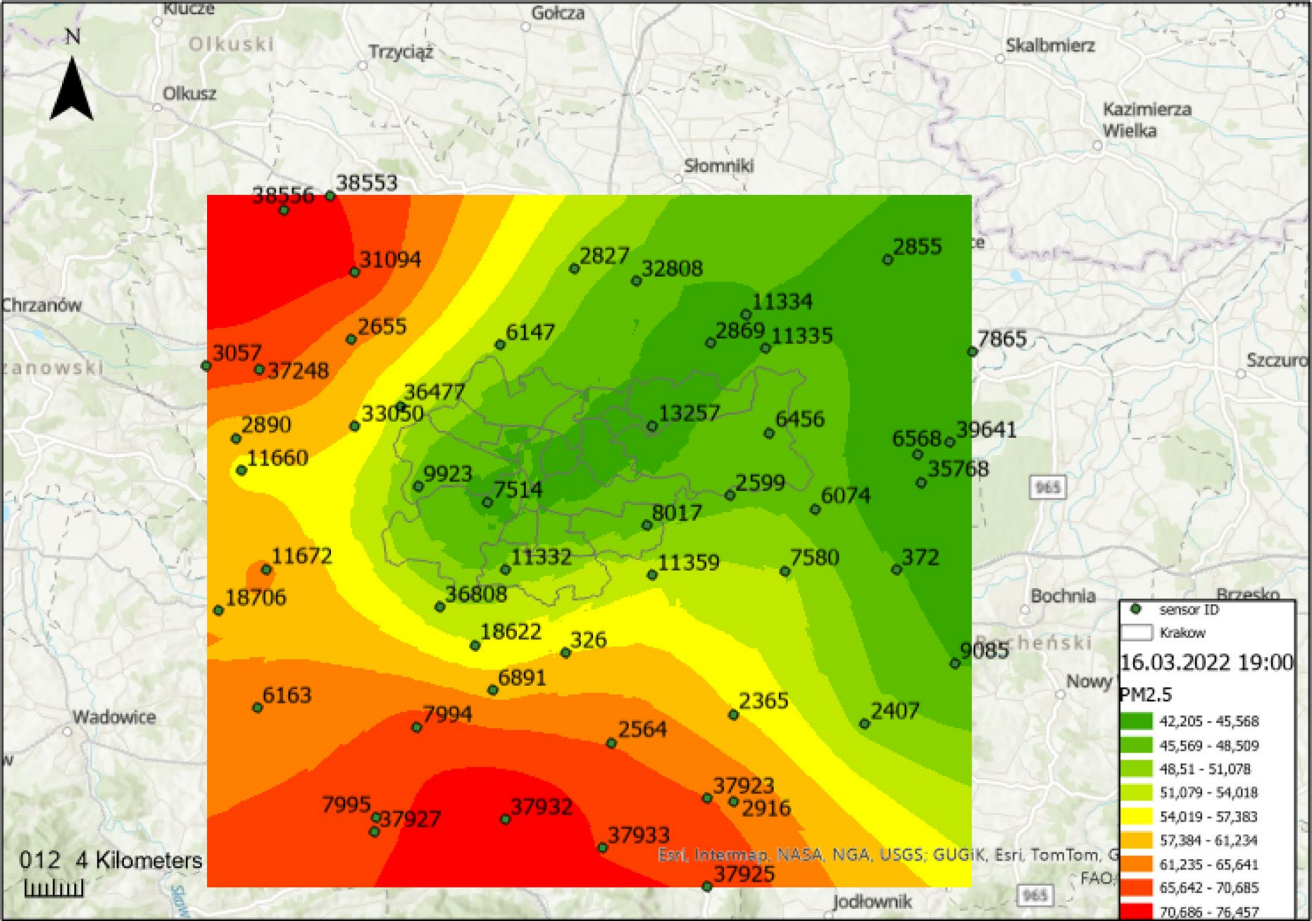
Spatial distribution of PM2.5 concentrations around Kraków area on March 16, 2022, at 19:00, calculated using the ordinary kriging method, own study (ArcGIS Pro 3.0.3, ESRI).

Figure 7 presents the PM2.5 distribution around Kraków area, calculated using the ordinary kriging method, depicting the concentration levels on March 16, 2022, at 19:00. The presented map clearly illustrates the spatial variability of PM2.5 concentrations in Kraków, with elevated levels observed on the periphery and lower concentrations in the city center.

When dealing with complex, massive datasets and attempting to model them, the first step should be to check for any underlying patterns that connect the data. PCA is particularly well-suited for this purpose, as it is highly effective in identifying correlated time series and uncovering hidden structures within the data.

**Fig. 8:**
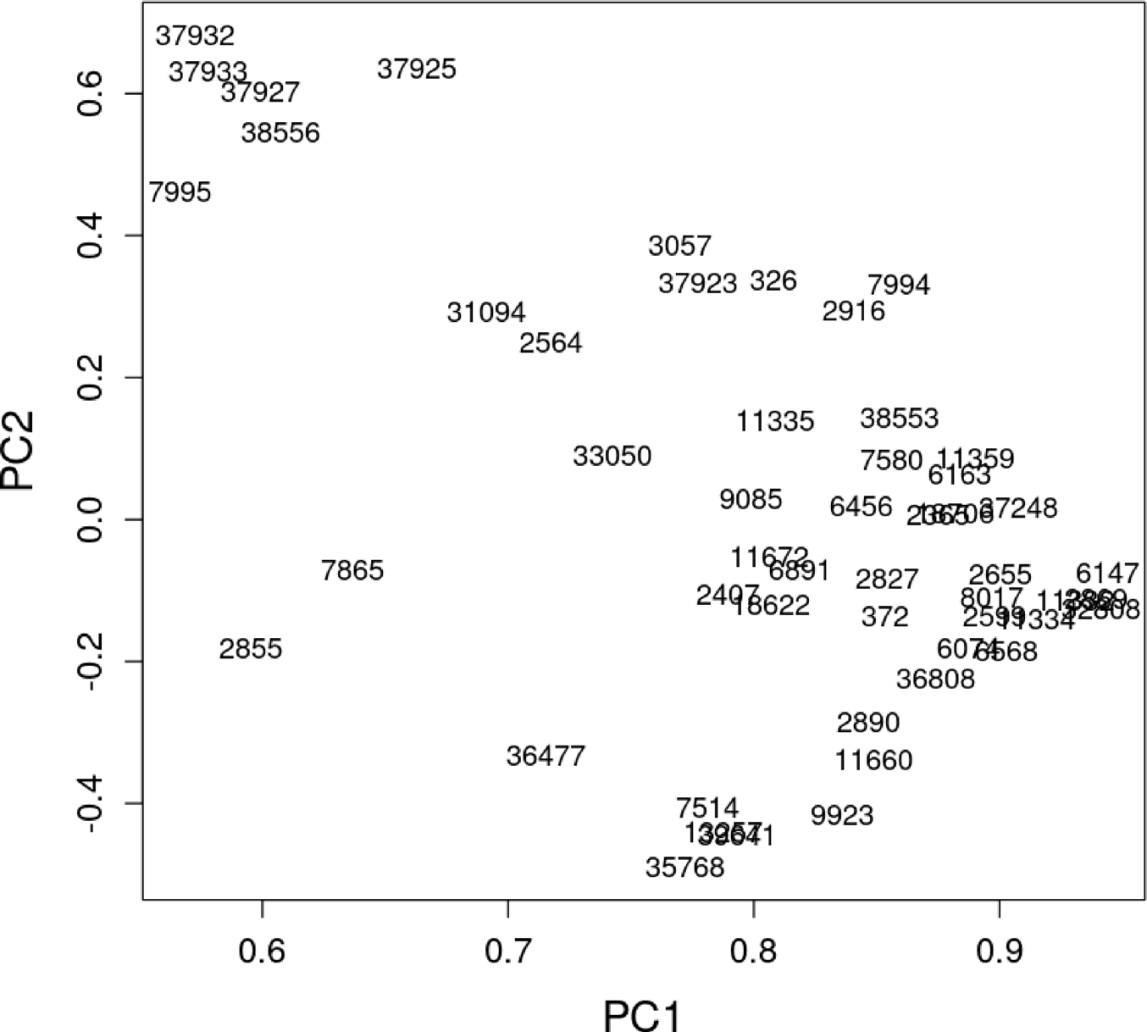
PCA results. Individual stations are marked by their IDs. The old furnace pattern group is clearly separated from other sensors in the upper right corner of the PC1/PC2 plane (compare Fig. 2).

**Fig. 9:**
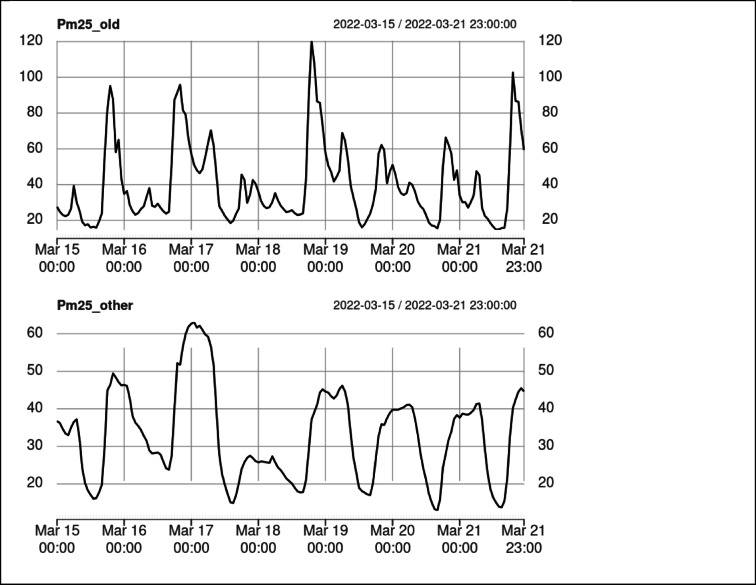
Average readings from sensors in the old furnace group compared to all other sensors.

The PCA results (Fig. 8) clearly distinguish a group of several sensors, specifically 37,932, 37,933, 37,927, 37,925, and 38,556 (compare Fig. 2). In Fig. 9, the averages obtained from both groups formed on PC1/PC2 plane are presented. A distinct drop in values during the late night is clearly visible within the data from the smaller, separated group. This strongly suggests that the measurements were taken near old, manually operated furnaces that do not receive fuel when the household members are asleep. These furnaces are generally responsible for increased emissions, as clearly shown on the vertical scale in Fig. 9. We will refer to this group as the “old furnace group,” while the remaining sensors will be referred to as the “other group”. It should be emphasised that this identification rests on indirect evidence (the diurnal concentration pattern) and therefore represents a high-probability conjecture and not a field-verified fact.

The models for the averages of both groups are examined next, with consideration given to the averaged weather parameters. Due to the circular nature of wind direction variables, these variables were appropriately transformed. A standard meteorological framework was applied, where wind direction and speed are expressed as wind speed separated as the northern and eastern components.

In the model creation step, RF^55^ models were applied. It was done due to their robustness and versatility. Given the inherent randomness of these models, slight variations can occur across different runs. To mitigate this, all calculations were repeated multiple times, and the final results were obtained by averaging after outliers were removed. RF models were trained using meteorological and temporal predictors. Model quality was assessed with the coefficient of determination (R2), because the amount of explained variance is critical for the subsequent XAI analysis. Obtained R2 exceeded 0.8 for every model. These high scores are consistent with the expected strong meteorological and time-of-day control on PM concentrations and confirm that the RF models provide an adequate explanatory basis for the later XAI steps.

In the first step, the importance of predictors was assessed for both series (old and other). The dataset included predictors such as: year hour (yh), day hour (dh), 2 m temperature (temp), relative humidity (hum), surface pressure (pres), precipitation (prec), cloud cover (cl), soil temperature (st), soil moisture (sm), wind speed in the eastern direction (ve), wind speed in the northern direction (vn), and weekday (wd). The first goal was to eliminate highly correlated variables, such as dew point and surface pressure. Predictor importance was measured using the permutation method, where the values of a given predictor are shuffled, leading to the loss of the original relationships in the data. The drop in model accuracy is then measured, indicating the importance of the predictor. Results of such analysis are presented in Fig. 9.

For the old furnace group, the main predictor was clearly the hour of the day. This indicates that pollution production in this group during the analyzed week, which had typical winter weather conditions for Poland, was entirely governed by an inhabitant’s behavioral pattern.

For the “other” group, the main predictor is humidity, which significantly surpasses the next most important predictor, temperature. Based on our model, we can hypothesize that pollution (probably at least partially incoming) is being “trapped” by moisture, captured from the air and kept close to the ground due to the increased mass of the particles. We interpret the role of humidity as the most plausible explanatory mechanism, yet without experimental validation. The presence of water molecules in aerosols also affects the PM2.5 measures, as the water content in aerosols ranges from 20 to 35% at a relative humidity of 50%^64^. It’s also worth noting that precipitation is another important predictor for this group. Additionally, it is worth noting that while humidity also plays a significant role for the old furnace group, it is not a critical factor.

Based on the H statistic^56^, the other important model detail, the interaction between predictors can be examined. The focus is not solely on the contribution of interactions to the influence of individual variables (measured as a percent of variance explained by a particular predictor) but rather on identifying which parameters exhibit the most impactful 2-way relationships with the main predictor.

**Fig. 10:**
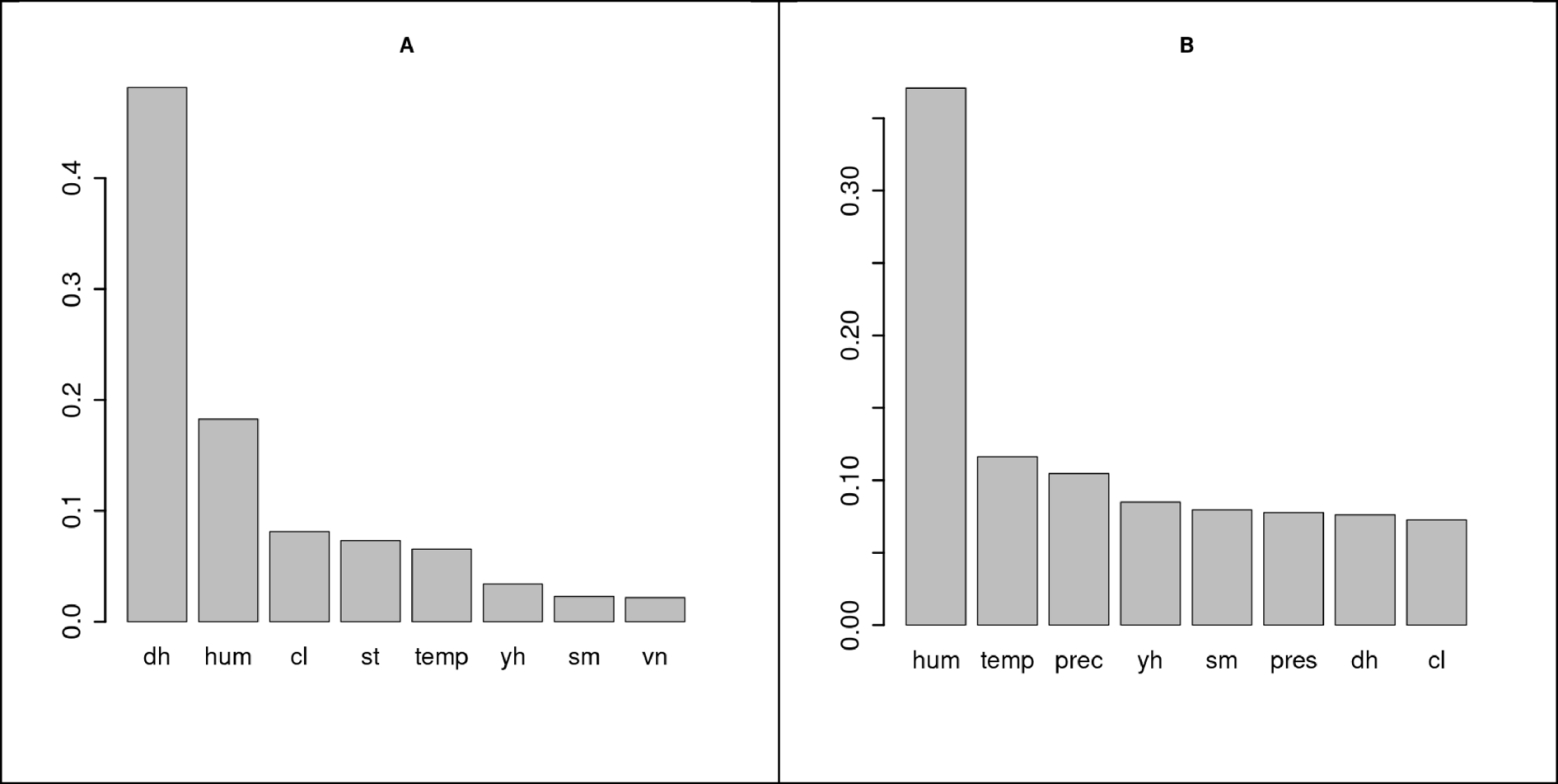
Predictor importance for the old furnace group (**A**) and the remaining sensors (**B**). See text for details.

Figure 10 shows that, in the case of old furnaces, the amount of variance explained by the primary predictor (hour of the day) is most significantly influenced by temperature. This indicates that their cumulative effect shapes the local level of pollution. In contrast, for the “other” group, the effect of interactions appears to be more random, and the interaction between the main (humidity) and other predictors is less significant. Nonetheless, the cumulative importance of humidity and temperature can be observed.

**Fig. 11:**
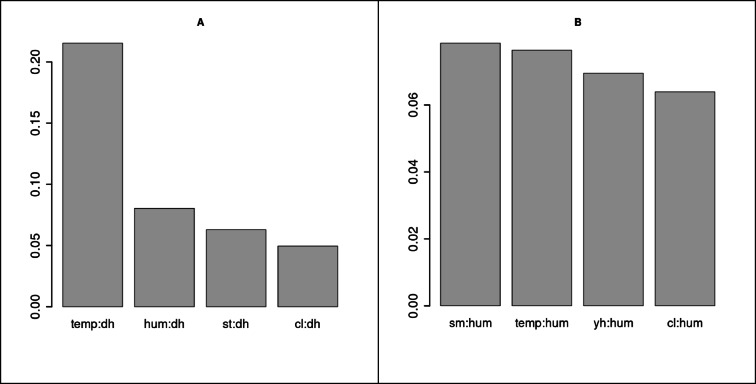
The interaction magnitude of the main predictor with other predictors for the old furnace group (**A**) and the remaining sensors (**B**). Result based on the H-statistic.

To further investigate the dependencies within the model, the Accumulated Local Effects (ALE) method^57^ was applied in the next step. ALE is used to interpret the influence of individual features on the model’s predictions, allowing for an understanding of nonlinear relationships between predictors and the model’s response. This method presents the accumulated effects of features on predictions, allowing the analysis of models with complex relationships. Figure 11 shows the results obtained for the main predictors in both groups, as well as for temperature.

**Fig. 12:**
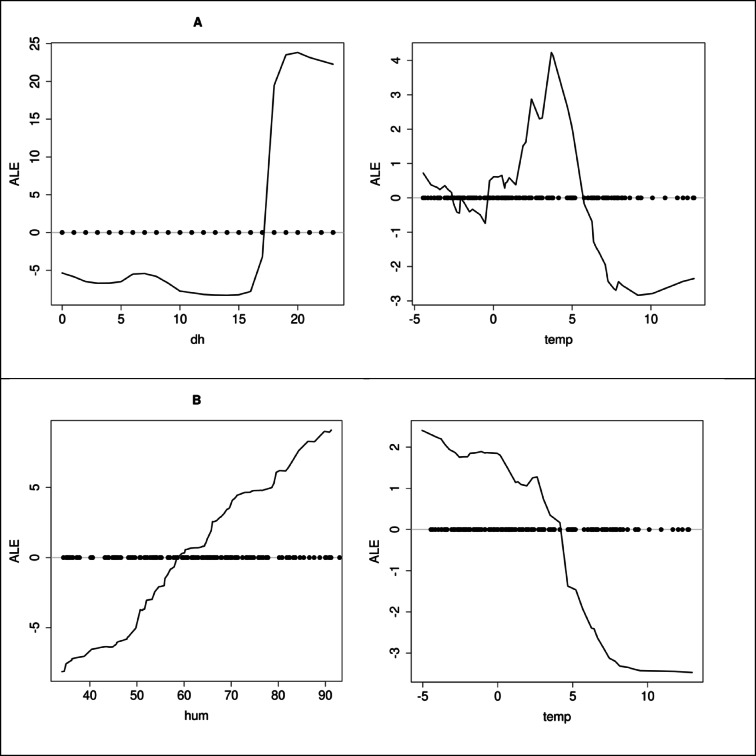
ALE results for both groups for the main predictors and temperature. Old furnace group (**A**), other sensors (**B**). The light gray line represents 0, with points on the line indicating the actual values of the predictors.

For the old furnace group, the influence of the main predictor (the hour of the day) is clearly nonlinear (Fig. 12). The key factor appears to be the start of heating in the afternoon, traditionally associated with people returning home from work. The observed pattern likely corresponds to the lack of automation in old furnaces. Regarding temperature, the impact of air temperature at 2 m transitions from negative to positive around 6 °C, reaching a peak at approximately 4 °C. It then decreases almost to zero, before slightly rising again for subzero temperatures. It’s important to note that during the analyzed period, the average difference between the apparent temperature and the temperature at 2 m was about 4.1 °C. This is consistent with previous findings, which indicate the significant role of the perceived temperature of 0 °C on PM2.5 emissions.

For the “other” group, the main predictor, humidity, has a linear impact on the observed PM2.5 levels, which seems to support the hypothesis of possible pollution “trapping” (Fig. 12). As for temperature, we observe a more regular influence of this predictor, with distinct inflection points in the nonlinear relationship at around 6 °C (where its significance increases) and around 0 °C (where its significance stabilizes). The transition from limiting to enhancing pollution occurs at approximately 4 °C. It is important to note that the cumulative effect on PM2.5 in this case is nearly half that observed for the old furnace group. The results for both humidity and temperature suggest that the pollution is being transported from external sources and that local emissions likely originate from more modern, automated furnaces.

It is important to note that the sensors recording the highest PM2.5 values and exhibiting the strongest patterns of emissions from old furnaces are not located within the so-called “bagel belt” surrounding Kraków but are instead situated further north (Jerzmanowice-Przeginia municipality) and south (Sułkowice, Myślenice, and Wiśniowa municipalities). While pollution originating from these areas may have some impact on air quality in Kraków, it is again important to consider the prevailing westerly winds in the region. Given this wind pattern, efforts to improve air quality in Kraków should prioritize municipalities directly to the west of the city that continue to have a high share of solid fuel heaters, particularly Czernichów and Skawina. These municipalities are likely to have a more significant impact on Kraków’s air quality and should therefore be a focal point for targeted interventions which corresponds with the results from previous section.

### Machine learning-based grouping of emissions

**Fig. 13:**
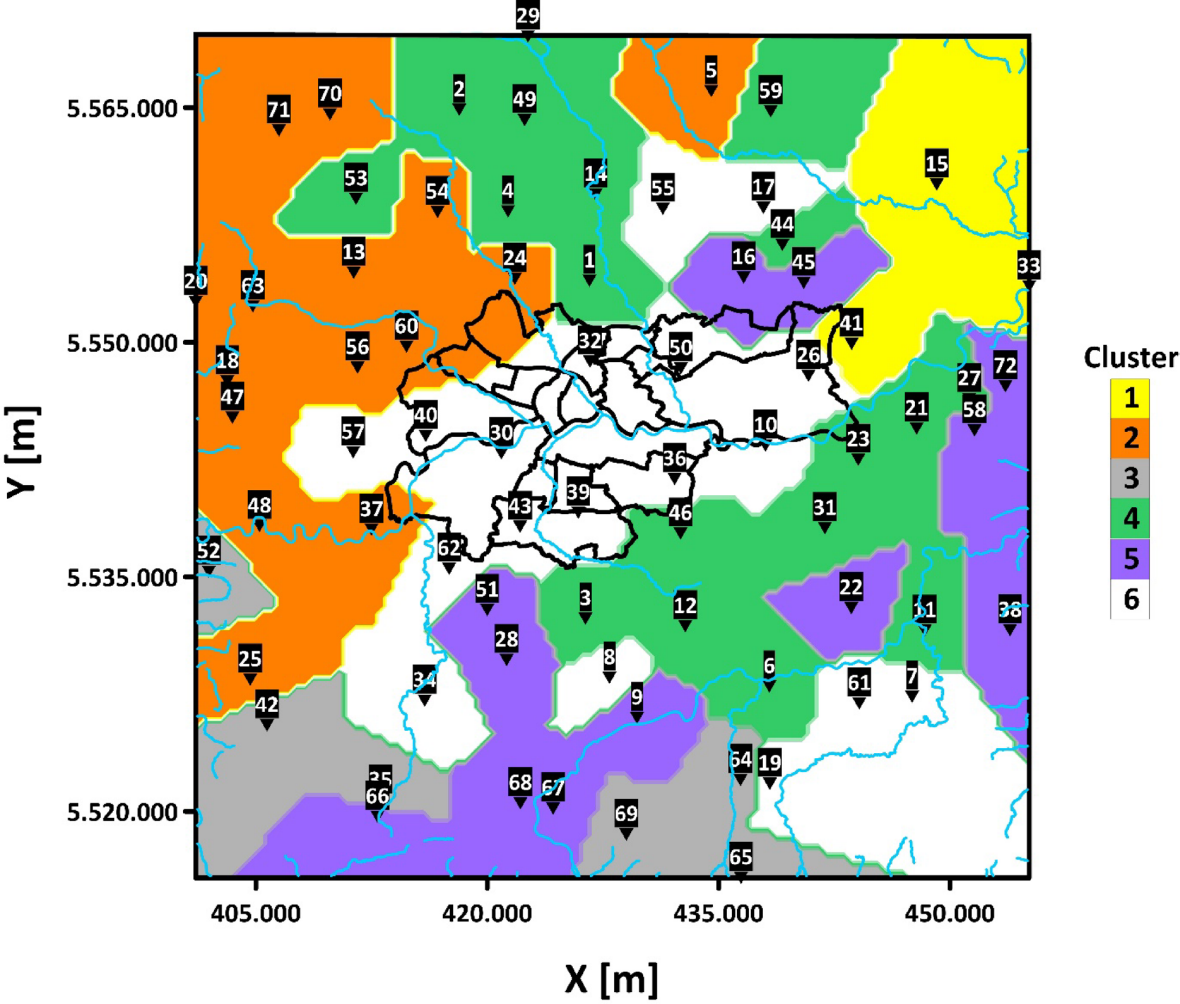
Map of PM2.5 Clusters (2019–2022) with LCS Locations (black boxes with white text), Rivers (blue lines), and Kraków City Districts (black boundaries), own study (Surfer 21.2.192, Golden Software LLC).

Among the distinguished two groups: “old furnace group” and ‘other group’, correlations can be observed related to the number of appliances and the dynamics of heat source replacement. Significant observations were made with the help of clusters, shown in Fig. 13. The unsupervised clustering revealed a clear distinction between Kraków and other municipalities, which may reflect the energy transition occurring in household heating. Clusters 3 and 5 highlight the complexity and heterogeneity of the “old furnaces” pattern, as shown in Fig. 2. Cluster 4 likely corresponds to areas with fewer old furnaces, possibly linked to higher income levels and the success of clean air policies.

**Fig. 14:**
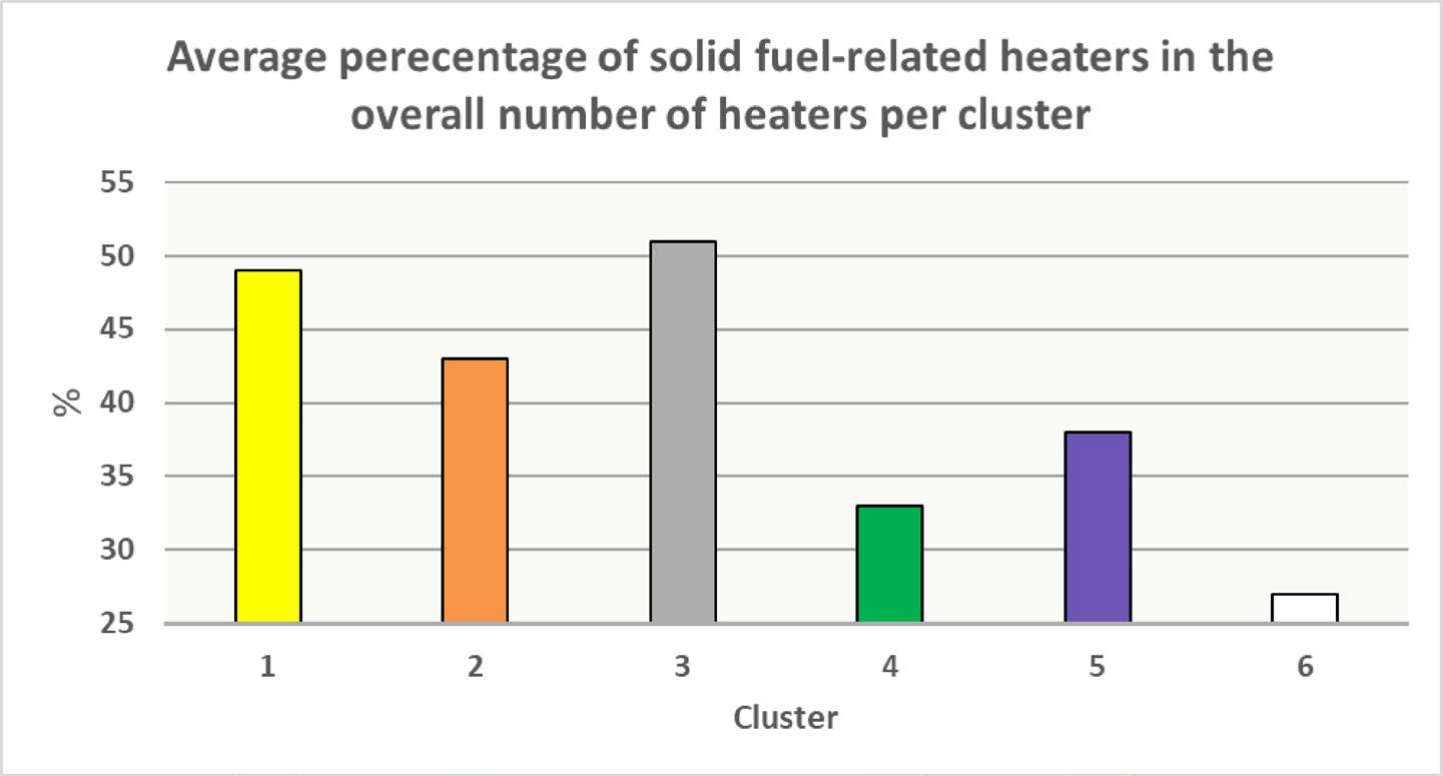
Average percentage of solid fuel-based heaters within total heater count across PMx clusters.

The average percentage of solid-fuel-based heaters (AHP) in the overall number of heaters per cluster is shown in Fig. 14. Data from cities and villages with fewer than 20 total heat sources were excluded to avoid skewed results, ensuring more reliable comparisons across clusters. Excluded LCS locations include LCS 8—Gołoszyn (8 total heat sources, THS), LCS 11—Pierzchów (16 THS), LCS 59—Waganowice (5 THS), LCS 72—Chobot (4 THS), LCS 45—Karniów (18 THS), LCS 7—Jaroszówka (14 THS), LCS 19—Kwapinka (4 THS), LCS 43—Prandocin (6 THS), and LCS 55—Zagorzyce Stare (7 THS). For Cluster 1, the AHP is 49%, with little variation between sensors. In Cluster 2, with an AHP of 43%, the highest percentage of “old furnaces” is in LCS 71—Czubrowice (62%), while the lowest is in LCS 13—Więckowice (25%). Cluster 3 has an AHP of around 51%, with the highest value being 76% in both LCS 64—Czasław and LCS 65—Wiśniowa. Clusters 4, 5, and 6 show much lower AHP values, ranging from 27% to 34%, with the lowest values in Cluster 6 largely representing Kraków. Notably, Kraków itself has an AHP below 5%, a direct result of legal restrictions on the use of fossil fuels.

As mentioned, in the case of the “other” group, the main predictor, humidity, has a linear effect on the observed PM2.5 levels, which seems to support the hypothesis of possible “capture” of pollutants. Table 3 shows the number of solid fuel stoves with automatic feeders, as well as their percentage in the number of solid fuel heating sources. The lower percentage of stoves with automatic feeders suggests a greater use of other devices, requiring manual operation of the equipment. The clustering results of PM concentration align with the spatial distribution of solid fuel heaters in Fig. 5, clearly showing that factors such as thermal efficiency and the type of heating source in buildings are directly related to the temporal and spatial distribution of air pollution in the region. This is a key observation, highlighting the need to integrate environmental protection with energy and urban planning to address the long-term issue of air pollution as exposure to PM leads to neurodegenerative diseases, which, in the context of an aging population, could pose a significant challenge to the healthcare system.

**Table 3:** Number of solid fuel stoves in selected municipalities and the share of stoves with automatic feeders as of December 31,2023.

Municipality	Number of solid fuel heat sources, as of 31.12.2023	Number of solid fuel stoves with automatic feeder, 31.12.2023	Percentage of automatic feeder stoves in all solid fuel appliances in municipalities (%)
Liszki	3486	642	18
Zabierzów	5615	825	15
Wielka Wieś	2687	356	13
Zielonki	3758	512	14
Michałowice	2543	339	13
Kocmyrzów-Luborzyca	3239	509	16
Koniusza	1809	383	22
Igołomia-Wawrzeńczyce	1618	426	26
Niepołomice	5227	501	10
Biskupice	2277	315	14
Wieliczka	9669	1103	11
Świątniki Górne	1995	272	14
Mogilany	3251	445	14
Skawina	5325	1301	24
Czernichów	4802	1256	26

## Conclusion

The analysis shows that in order to ensure adequate air quality in Krakow, a restrictive policy limited to the city limits is insufficient, and measures extended to neighbouring municipalities are urgently needed. The air protection policy, which led to the ban on the use of solid fuels in Krakow, was based on public funding, mainly from the EU, for the replacement of equipment. In a large city, this was facilitated by the fact that a large proportion of households could use the municipal heating network. In addition, a protective programme was provided for the poorest residents. In the case of municipalities neighbouring Krakow, support under programmes for individual residents is one-off, which means that beneficiaries calculate that the heat source they install will later be relatively cheap to operate. Residents can take advantage of various support programmes, but these often do not cover a small part of the investment in cases where households need comprehensive support, including thermal modernisation. In other cases, replacing the heat source with a less polluting one without comprehensive modernisation may result in a significant increase in bills for many residents.

According to the authors, one-off support, limited to the duration of the programme/project, may prove insufficient. Therefore, it is crucial that programmes are targeted, reach those most in need of assistance, and focus on areas that will have a positive impact on the health of the entire community. Our convincing machine learning (ML) models show that effective implementation of knowledge-based management is possible because all important patterns have been clearly demonstrated. In our opinion, more comprehensive solutions are needed for air quality improvement measures, going beyond the project/programme formula and fitting into the sustainable development paradigm^65^. One of the sustainable development goals defined by the UN explicitly points to the need to improve air quality in urban areas^66^. Current programmes of Polish local governments do not fully fulfil their role, partly due to a lack of continuity.

The authors argue that in order to improve air quality in metropolitan areas, regional authorities should standardise heat source replacement policies across municipalities and introduce financial mechanisms to support economically disadvantaged populations. In addition, decision-making processes should take into account spatio-temporal analysis to reflect the specific nature of pollution in individual regions. Without such comprehensive solutions, it will be difficult to implement effective policies focused on sustainable development. Such a sustainable policy of supporting residents is possible thanks to the precise data we provide in our analysis. Administrations have even more precise information at their disposal to identify households in need of additional assistance. According to the information we have provided on the wealth of municipalities, a correlation can be demonstrated between the number of solid fuel heat sources and the tax revenues of local government units. This is an important indicator in the context of such measures. Among the municipalities analysed, Czernichów and Skawina stand out as critical areas due to the high proportion of solid fuel heating devices, economic conditions and the direct geographical impact on Krakow. According to the authors, addressing the issue of emissions in these municipalities should be a priority in order to improve air quality in the city.

The case study from the Krakow agglomeration may indicate the need to modify support in the form of programmes/projects to a more systematic and targeted form. This can be called ‘sustainable support,’ which should go beyond time limits. The model we’ve presented shows exactly which types of devices cause emissions, as well as predictors related to their use. ML analysis of spatiotemporal data on PM2.5 revealed clear patterns associated with the low number of old furnaces in Krakow and identified an increased emission cluster outside the city, which likely highlights the interaction between air quality and socio-economic factors. Please note that these findings, though robust, are drawn from a single mid-March week of observations and from low-cost sensor records that inherit the usual measurement and gap-filling uncertainties. Even though these limitations might affect the details of the results, the main patterns we found stay clear. The analysis shows that the use of solid fuels as heat sources determines air quality in Krakow. The authors believe that in order to change this situation, it would be necessary to switch to non-emitting sources throughout the Krakow agglomeration.

## Data Availability

Publicly available datasets from Airly sensors were analyzed in this study and can be found here: (https://map.airly.org/, accessed on 17 Feb 2022). API documentation from Airly is available here: (https://developer.airly.org/en/docs, accessed on 17 Feb 2021). Publicly available datasets from the Chief Inspectorate For Environmental Protection database were analyzed in this study. This data can be found here: (http://powietrze.gios.gov.pl/pjp/home, accessed on 17 Feb 2022). API documentation is available here: (http://powietrze.gios.gov.pl/pjp/content/api, accessed on 17 Feb 2022). Information on the number of heat sources was provided by the General Office of Building Control in Poland. The database is not accessible to the public, but may be made available to research units indicated in the Law on Higher Education and Science for scientific purposes upon reasonable request. For data inquiries, please contact Maciej Sabal ([sabal@agh.edu.pl](mailto: sabal@agh.edu.pl) ).
